# Executive Functions and Domain-Specific Cognitive Skills in Climbers

**DOI:** 10.3390/brainsci11040449

**Published:** 2021-04-01

**Authors:** Florian Heilmann

**Affiliations:** Movement Science Group, Institute for Sport Science, Philosophical Faculty II, Martin-Luther University Halle-Wittenberg, 06108 Halle (Saale), Germany; florian.heilmann@sport.uni-halle.de; Tel.: +49-0345-5524554

**Keywords:** cognitive functions, executive functions, skills transfer, climbing, working memory, inhibition, cognitive flexibility

## Abstract

Athletes in a particular sport have specific cognitive skills acquired due to regular confrontation with sport-specific requirements. Studies show that the particular type of sport carried out and fostered by general physical activity impacts executive functions (EFs) such as inhibition, working memory, and cognitive flexibility. There are inconsistent results on the connections between domain-specific cognitive skills and executive functions. This study aimed to evaluate the relations between EFs and domain-specific cognitive skills in climbing. Due to that, we examined the executive functions (neuropsychological tests) and domain-specific cognitive skills (climbing-specific test: a preview of the route vs. climbed moves; climbed moves vs. recognition of moves) of 19 climbers (10 novices, 9 experts, grades 5 to 6a vs. 6c+ to 7b). The inter-subject effects analysis shows that novices and experts in sport climbing do not differ in executive functions in this particular case. Concerning domain-specific cognitive skills, there are differences between experts and novices. Experts show a significantly higher level in planning performance or route idea (*p* < 0.001) as well as in memorizing of climbed moves (*p* = 0.004). There are no relations between executive functions and domain-specific cognitive skills in climbers.

## 1. Introduction

Athletes with special movement experience seem to be superior in cognitive tasks or process requirements to people with less movement experience (for reviews, see [1,2,3]). This advantage could be attributed to general physical activity, aerobic fitness level [4], or the habitual effects of sport and physical activity [5]. Furthermore, the execution of specific sports with movement experiences typical of the sport type promotes expert performance in cognitive skills or executive functions [6,7,8]. There are direct connections between cognitive skills or executive functions and sport-specific motor skills reported for soccer [9] and volleyball players [10]. Furthermore, the links are studied in the field of climbing.

### 1.1. Connections of Executive Functions, Cognitive Skills, and Climbing

“Executive functions (EFs) make possible mentally playing with ideas; taking the time to think before acting; meeting novel, unanticipated challenges; resisting temptations; and staying focused. Core EFs are inhibition (response inhibition (self-control—resisting temptations and resisting acting impulsively) and interference control (selective attention and cognitive inhibition)) working memory, and cognitive flexibility (including creatively thinking ‘outside the box’, seeing anything from different perspectives, and quickly and flexibly adapting to changed circumstances)” [11] (p. 135). These functions are the basis for higher-order EFs such as reasoning, problem-solving, and planning. In contrast, domain-specific cognitive skills are related to the explicit affordances of a type of sport. For example, soccer requires great visual–perceptive and anticipation skills [12].

Climbing supports essential cognitive and psychological skills [13,14]. Before a climber starts a challenging route, he perceives the particular route’s affordances and plans the actions to perform at the wall (route finding [15], planning, problem-solving). The climber has to perceive the hold’s reachability from various positions [14] and its grip characteristics and decide if the hold can be grasped or used for foot support [14,15,16]. Planning (problem-solving and planning) and remembering (working memory) routes are relevant cognitive skills and EFs in climbing, which means that climbers have to solve the “problems” to be able to climb the routes.

Previous findings [17] describe that deciding on the wrong path or the wrong move makes a major difference in climbing performance. A false movement caused by the perception of competing information or a competing attraction can lead to a fall. For this reason, climbing athletes must be well trained in inhibiting competing stimuli (inhibition).

However, if an error occurs in the route planning or the athlete realizes that the route cannot be completed, he must change his strategy. The skill to change plan and adapt to change in perception could be described as cognitive flexibility. The expert’s performance should be verifiable in general executive function tests and domain or sport-specific examination, and they should show a retainable connection. There are results for and against the relationships in previous research.

Chase and Simon [18] demonstrate that chess players do not have better test results for working memory than a control group of non-chess players. Huijgen et al.’s [19] findings could also report no differences in working memory capacity in higher- and lower-level soccer players. Contrasting results [20] show significant differences among soccer players. Whitaker et al. [21] examined the working memory capacity in climbers using the Corsi block test. In their study, skilled climbers did not outperform less experienced climbers. They postulate that expert climbers could chunk visual information on a climbing route into a manageable number of parts, similar to chess experts [18,22]. The skills are characterized as domain-specific. Boschker et al. [14] also experimented with climbers. Participants had to reproduce the locations and orientation of 23 holds of a climbing wall. Expert climbers recalled more and clustered information and focused on the climbing wall’s functional aspects and ignored the route’s structural features. Climbing novices did not recognize such information and reported almost exclusively the holds’ structural features [14]. So, there are inconsistent results regarding the difference in experts and especially climbers in working memory capacity.

Results of Cascone et al. [23] are connected to the hypothesis that climbers outperform non-athletes in inhibition or executive functions. The results show that climbers with advantages in climbing sports score better in the Tower of London test (EF).

Wickens et al. [24] postulated that outdoor rock climbing is a multi-task activity in which the climbers have to switch attention between tasks. The task affordances given in climbing sport could result in advantages in task-switching performance or the athletes’ cognitive flexibility compared to non-athletes.

### 1.2. Transfer of Cognitive Skills

Numerous findings document the differences in cognitive skills and especially EFs between elite athletes and non-athletes playing tennis [25], volleyball [26], baseball [27], basketball [28], and soccer [9,19]. The differences are often explained by cognitive skill transfer (CST). The process describes that cognitive skills in a cognitive task could improve a related untrained cognitive task. Moreover, training in a specific environment could improve athletes’ cognitive skills [9,19]; the CST model agrees with the assertion that every task affordance consists of different skills and/or specific knowledge.

The model is often discussed concerning the “expert performance approach”, which describes that athletes have improved cognitive abilities only within their sport [29]. The second and contrary approach—the “cognitive component skills”—assert that athletes improve their sport-specific cognitive skills, which are transferred or also present in various non-sport contexts and could lead to advantages in laboratory measures [2].

The inconsistent results about the transfer of cognitive skills in athletes lead to the research question in the scope of how “far” the skills could be transferred. Concerning the currently researched climbers’ population, the question is how “far” participants’ cognitive skill is transferred to the laboratory task.

Findings of Chase and Simon [18] revealed that chess players appear to have superior working memory regarding chess pieces’ arrangements, but their overall working memory is normal. The findings support the “narrow transfer” hypothesis because the chess players as experts in their field show superior cognitive processes in that field, but not outside. Contradictory results show improvements in reaction time tests caused by extensive video game training [30]. The “broad transfer” hypothesis postulates that the practice of context-specific skills improves individual components of cognition or cognitive skills regardless of the context [31].

It remains unclear if the cognitive skill advantages in a particular sport are only domain-specific or if experts outperform novices in domain-unspecific tests.

The present study aims to clarify for the first time the extent to which the climbing athletes’ cognitive skills can be viewed as domain-specific (measured in the field) or as general cognitive skills. To examine the relation between domain-specific cognitive skills and essential cognitive functions such as executive functions (inhibition, working memory function, cognitive flexibility), a cross-sectional design with EF tests (Stroop Color Word Test (SCWT), Corsi-block test (CBT), digital Trail Making Test (dTMT) and Wisconsin Card Sorting Test (WCST) was conducted. The participants were further tested in the skill to predict what moves they would make to climb the route (two routes) and to remember the moves they did at the wall (domain-specific skills).

The results should lead to a theoretical description of the relations and practical implications for cognitive training in everyday life. Suppose there is a connection between general and domain-specific skills. In that case, climbing training could be used as a training tool for cognitive skills, and the reverse is also conceivable—athletes could use cognitive training to improve their climbing performance. The results could lead to further information for the debate about the “narrow” and “broad” transfer hypothesis.

We hypothesize that the experts in climbing outperform the novices in the (a) test for EFs and the (b) domain-specific cognitive examinations (predicting and remembering the climbing moves). The central hypothesis is that there is a relation between EFs and domain-specific cognitive skills (c).

## 2. Materials and Methods

### 2.1. Participants

A priori power analysis (G*Power, version 3.1.9.6) was used to calculate the study’s required sample size. The results of Pietsch and Jansen et al. [32] were used to calculate an optimal sample size for the executive functions tests. The results of Boschker [33] served as a basis for planning the analysis for domain-specific cognitive tests. For the first analysis, the difference between two independent group means (two-tailed test) and the effect size from these results of Pietsch and Jansen [32] were used (2018; *d* = 1.89; M_1_ = 14.06; M_2_ = 10.8, SD_2_ = 2.10 α = 0.05). The result showed that a total sample of 14 participants with two equal-sized groups of *n* = 7 was required to achieve a power of 0.95. The reported differences show a significant effect between experts and novices in the mental rotation test (domain-unspecific test). For the second a priori power analysis, the difference between the two independent groups’ means using an ANOVA (omnibus, one-way), an effect size from the results of Boschker et al. ([33], *d* = 1.28; M_1_ = 38.00; M_2_ = 47.00), and an alpha of 0.05 was implemented. The result showed that a total sample of 12 participants with two groups of *n* = 7 was required to achieve a power of 0.95.

Nineteen climbers (9 females, 10 males) between the ages of 18 and 31 (M = 24.15, SD = 3.66) were recruited with a flyer and online call from a local climbing gym. The 19 climbers gave their written informed consent to participate in this study. Participants were compensated with a voucher for the climbing gym for their participation.

Mean height of participants was reported as 174.2 cm (SD = 7.03 cm) and mean body weight 68.6 kg (SD = 11.67 kg). The participants self-reported skill levels (reported in French scales) ranged from 5–6a to 6c+–7b (M = 6a+). The training hours reached from 1 to 5 h (M = 2.10, SD = 0.10). The climbers were divided into two groups based on their self-reported skill level (10 novices, 9 experts). There were no differences in weight, height, or age between the two groups (see Table 1). These results legitimize the investigation of the differences in cognitive functions. The differences could not be influenced by cofactors, such as age or anthropometrical data. The two skill groups differed in training hours per week (1.6 vs. 2.7 h/week; *p* = 0.030).

### 2.2. Measurement of Executive Functions

The study of the analysis of cognitive skills contains four various neuropsychological tasks. The tests indicate participants’ performance concerning inhibition, working memory, cognitive flexibility with Stroop Color Word Test, Corsi block test, digital Trail Making Task, and Wisconsin Card Sorting Test.

**Figure 1:**
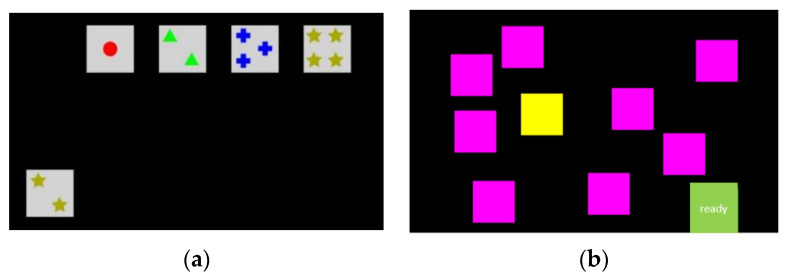
Stimulus presentation for Wisconsin Card Sorting Test (**a**) and Corsi block test (**b**).

The Stroop Color Word Test (SCWT, Ref. [34]) is an experimental psychological method for testing cognitive processing conflicts. The subjects have to name the colors of the words presented. If the word displayed that describes the color (color word) does not correspond to the font color, the reaction time and the number of errors increase (Stroop effect, incongruent stimulus). The test evaluates the ability to inhibit competing information (color word). In this study, the correct color was selected on the keyboard with colored keys (Figure 1). The stimuli (32 words in total, the delay between stimuli: 500 milliseconds) were displayed for 2 s. The reaction and action time (congruent (rtcon), incongruent (rtincon)) as well as the Stroop effect (rtinconc-rtcon) were calculated. The Corsi block test (CBT, [35]) examines the function of visual-spatial working memory. The test person had to memorize the sequence of squares shown in yellow and had to reproduce them. The difficulty increased after a correctly completed pass due to the number of squares to be memorized. The squares were pointed out with the mouse pointer. The number of squares memorized in the correct order was used to describe working memory (span).

Participants completed the digital Trail Making Test (dTMT), which was shown to be a valuable test for measuring executive function [36]. The test examines how fast the participants connected 25 circles with numbers (test run: 5 circles) by drawing a line. There were circles with nothing written or illustrated inside them, and circles with distracting patterns that had to be avoided by the participants. The task in Part B was to connect digits and letters (1-A, 2-B…) until they reached the number 13. Fifteen empty distractor circles appeared on the same page. There was a practice trial (sequence 1-A, 2-B, 3-C, 4-D, 5). The total time for Part A and B was measured. Part A is associated with visual search and processing speed [37]. dTMT Part B is characterized as a test exanimating processing speed and more complex cognitive skills such as cognitive flexibility and inhibition [38].

The Wisconsin Card Sorting Test (WCST, [39]) is a neuropsychological tool used to measure cognitive flexibility. The test contains four stimulus cards with geometric figures that have to be sorted according to a rule that the participants should recognize based on the feedback from the program (Figure 1). The sorting rule changed in the course of the test. Sixty-four cards were presented to the test person. The test can evaluate the limited ability to adapt, insufficient learning from feedback, and perseveration tendencies. In the study, the participant selected the appropriate card with the mouse pointer (computer-aided version). The total number of errors, perseveration errors, and errors that did not result from the strategy change was calculated.

**Figure 2:**
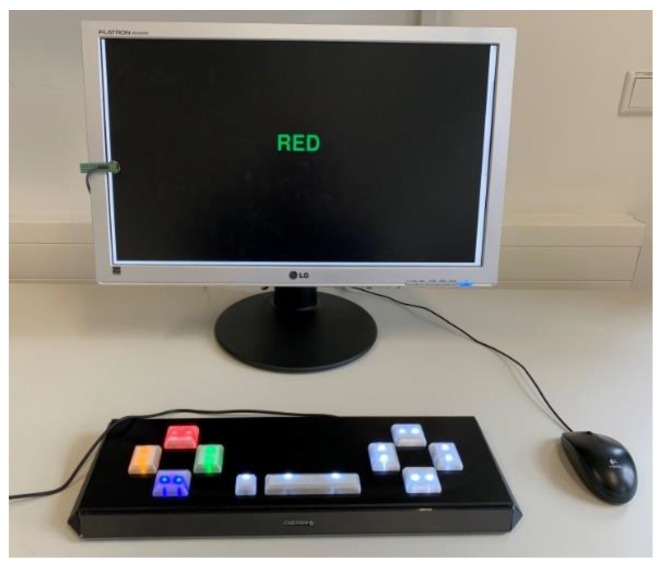
Measurement setup with the display, mouse pointer, and test keyboard.

The cognitive, neuropsychological computer tasks were carried out with the mouse pointer of a computer mouse (WCST, CBT, dTMT) or a specially adapted keyboard (Cherry GmbH, Auerbach in der Oberpfalz, Germany, Cherry KC 1000; in-house development; delay time: <5 ms; Figure 2; SCWT) and with the Software PsyToolkit [40,41].

The four triggers were arranged at the same distance from the center (crosses on the right and left) to ensure comparable action times. After the instructions had been presented on the 19-inch PC monitor, the test subjects started the tests independently. There was a two-minute recovery break between the four neuropsychological computer tests.

### 2.3. Measurement of Domain-Specific Cognitive Skills

**Figure 3:**
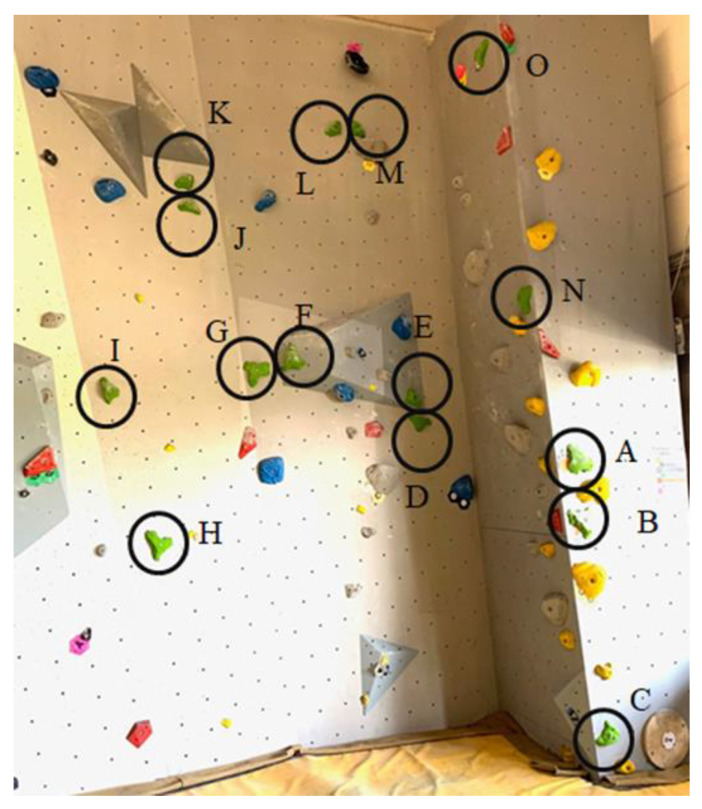
The second route in the domain-specific cognitive test (climbing test) with marked grips and holds (letters A–O).

With the domain-specific test, two parameters were calculated: the skill to predict the right moves to execute for climbing the route (1) and remembering the moves they had done in their climb (2). The first test evaluates a domain-specific skill of planning or pattern recognition in participants. The second test assesses the specific working memory function. The participants had to climb two routes with two different grades, which were not climbed any time before. The levels were not shown with colors at the start holds as usual. Grades were challenging for the selected population because Pezzulo et al. [42] only presented differences in cognitive skills between experts and novices if the tested routes were difficult enough (in line with the expert’s skill level). The routes corresponded to the average skill level of the two groups (6a+). After neuropsychological computer tests had been carried out, the participants had 10 min to warm up individually (for the climbs). Each participant had one minute to preview the route and was allowed to touch all the holds that could be reached in a standing position. After the preview, the participants had to verbalize their climbing idea (route finding) precisely so that the investigator could note every climbing move (hands and feet) on a form with a photo of the route (Figure 3). After verbalizing the route idea, the climber had the time to prepare for the climb (shoes and magnesium on the hands).

### 2.4. Statistical Analysis

To analyze the differences in the two groups’ cognitive skills (novices, experts), central tendency tests were carried out. A Shapiro–Wilk test was calculated to check the normal distribution of variables. Outliers were excluded when the value exceeded 1.5 IQR. Values did not exceed the given IQR. Values for 15 out of 20 variables were not normally distributed. Due to the non-normal distribution of the data, non-parametric tests were calculated to evaluate the differences and correlations. Differences between the groups were calculated using the Mann–Whitney U test (α-corrected with Bonferroni). The correlations between the tests were verified using the Spearman rho correlation (α-corrected with Bonferroni). Statistical calculations were executed using IBM SPSS Statistics 25 software.

## 3. Results

### 3.1. Executive Functions

The measurement of executive functions using the neuropsychological computer tests revealed no detectable difference between the experts and novices. In the Stroop Color Word Test, novices showed a longer duration for congruent and incongruent stimuli (novices: 731.00 ms; experts: 706.44 ms; novices: 700.30 ms; experts: 695.11 ms) as well as for the Stroop effect (relation of congruent and incongruent stimuli; novices: −58.30 ms; experts: −16.67 ms), but the differences were not significant (congruent: *U* = 29.00, *p* = 0.211, *d* = 0.49; incongruent: *U* = 43.00, *p* = 0.905, *d* = 0.08; Stroop effect: *U* = 27.50, *p* = 0.156, *d* = −0.79; Table 2).

The span for the Corsi block test differed between a mean value of 6.50 objects for the novices and 5.33 objects for expert climbers. The novices in climbing memorized significantly more objects than experts (*U* = 16.00, *p* = 0.017, *d* = 1.52). The time for the execution of the digital Trail Making Test Part A and Part B (Table 2) was not significantly different for novices and experts (Part A: *U* = 42.00, *p* = 0.842, *d* = 0.11; Part B: *U* = 44.00, *p* = 0.968, *d* = −1.00). Total errors (novices: 10.60; experts: 9.33; *U* = 28.00, *p* = 0.182, *d* = 0.34), preservation errors (novices: 6.80; experts: 6.56; *U* = 32.50, *p* = 0.315, *d* = 0.11) and non-preservation errors (novices: 3.80; experts: 2.78; *U* = 36.00, *p* = 0.497, *d* = 0.45) in the Wisconsin Card Sorting Test showed no differences between expert and novice climbers.

### 3.2. Domain-Specific Cognitive Skills

**Figure 4:**
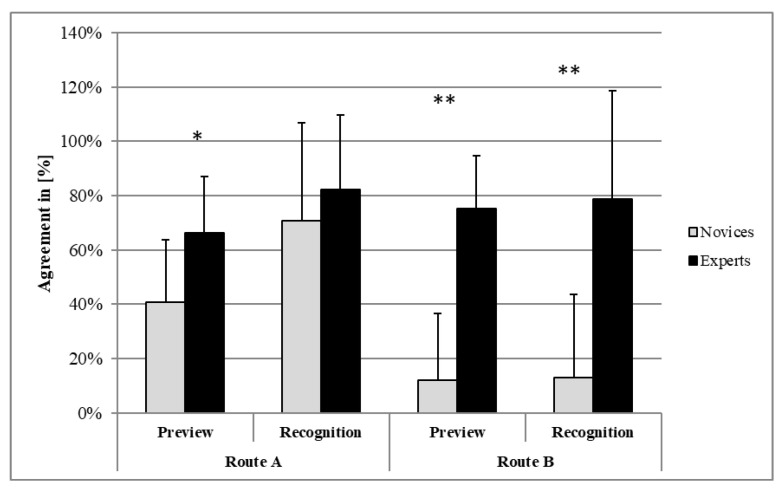
Percentage of agreement between the preview of the route and the climbed moves (preview) as well as the agreement of the climbed moves and the recognition of climbed moves (recognition); group differences were calculated by Mann–Whitney U test (* = *p* < 0.05, ** = *p* < 0.01).

The comparison of domain-specific cognitive skills between the two groups of climbers with different expertise levels showed significant differences in the planning (preview) and memorizing (recognition of the holds and grips) of the athletes (Figure 4). The agreement of the route idea and the climbed moves for the first route was higher in experts than in novices (preview: *U* = 16.00, *p* = 0.017, *d* = −0.79). The congruency of the climbed moves and the recognition of climbed moves did not differ between the two groups (novices, experts; recognition: *U* = 38.50, *p* = 0.604, *d* = −1.23) for the first route. In the preview and recognition of the second route, the experts showed significantly better performances than the novices (preview: *U* = 4.00, *p* < 0.001, *d* = 0.37; recognition: *U* = 11.50, *p* = 0.004, *d* = −1.96). The effect sizes of different measures were large.

### 3.3. Relation between Executive Functions and Domain-Specific Cognitive Skills

There were no noteworthy correlations between the values measured for executive functions and domain-specific cognitive skills. The only significant correlations between parameters of the tests for executive functions relate to the same test. For example, the value for total errors in the WCST correlated with the non-preservation errors and the preservation errors of the same test (rs(19) = 0.553, *p* = 0.014; rs(19) = 0.937, *p* < 0.001).

## 4. Discussion

The current study assesses the relations between the EFs and domain-specific cognitive skills in their entirety for the first time. The results show a significant difference in domain-specific cognitive skills between climbing novices and experts. There were no traceable connections between the test results for executive functions and the domain-specific tests for cognitive skills in climbing.

The study results confirm the findings of Whitaker et al. [21] that expert climbers do not outperform less skilled climbers in working memory capacity measured by the Corsi block test. There is no explanation why athletes with a lower level in climbing performance should have better working memory ability than the experts in climbing. Whitaker et al.’s [21] theory that expert climbers could chunk visual information on a climbing route into smaller manageable parts seems plausible. It indicates that the skill is characterized as domain-specific. They explain that advantages in sequence planning and memory performance are “likely not due to superior general visuospatial working memory, but to domain-specific ability” [21] (p. 317). The results show that climbing experts can memorize executed moves better than novices. These findings agree with Boschker et al. [14] that climbing novices did not recall much information and reported almost exclusively the holds’ structural features.

Boschker et al. [33] showed that climbers use route finding predominantly to practice the order of climbing movements (a kind of climbing choreography) to determine the best route, improve climbing performance, and find places to rest at the climbing wall (problem-solving and planning). The climbers get to know that route-finding mistakes are a significant reason for falling during climbing or bouldering. The findings of Boschker et al. [33] suggest that climbing skills might be related to climbers’ route-finding skills [14]. They have special domain-specific cognitive skills in pattern recognition and route-finding [14] or decision-making processes. The current study could support these results, but considering the working memory capacity, there could not be reported differences between novices and experts in the laboratory task. There are contrary results for the recognition task. Experts showed superior skills in remembering moves and grips in the domain-specific task. This finding supports a “narrow” transfer of cognitive skills because climbers could show superior cognitive skills in their domain but could not transfer the skills to a laboratory task.

Regarding the subjects’ ability to inhibit, there could be reported no differences between novices and experts, neither in the action and reaction times for the two types of stimulus presentation (congruent, incongruent) nor the relationship between these two variables (Stroop effect). This result could be explained by the importance of inhibition in climbing. Working memory may have a more significant impact on climbing performance than inhibition [21]. Another explanation is the domain-specificity of cognitive skills in climbers. Expert climbers may outperform novices in fading out irrelevant or competing information while climbing but not in a laboratory test. It seems that the ability to inhibit competing information could be examined only by neuropsychological tests. It is difficult to relate the construct of inhibition to the domain-specific tests in climbing. The results stand in contrast to Cascone et al. [23], who found that expert climbers show higher EF scores. They interpret climbers’ superiority in the Tower of London test as an expected greater impulsivity of climbers in challenging the climbing wall [23]. It must be stated that the test is not intended only to determine the construct of inhibition but rather the problem-solving ability and general executive functions.

Wickens et al. [24] hypothesized that outdoor rock climbing is a multi-task activity. The hypothesis that task affordances given in climbing sport could result in advantages in task-switching performance, or cognitive flexibility could only be declined with the current study results. There were no differences between novices and experts in WCST.

Although we controlled for age and differences in height or weight, we did not control for covariates such as IQ [43] or other activities that could be a contributor to EF measures (for example, playing musical instruments [44]). Furthermore, we did not control participants’ motivation, which has been shown to be a contributor to EF performance [45]. Due to the cross-sectional study design, we do not lay claim to a causal relationship. The test choice could always impact study results. The used tests were validated to examine working memory, inhibition, and cognitive flexibility. A lack of differences in climbers’ cognitive skills could be caused by the homogeneity of participants of the current study. Despite novices and experts being examined, the variance in climbing experience respectively vs. the climbing performance was slight (training per week: SD = 1 h; climbing grades: 5 to 6a vs. 6c+ to 7b). The analysis of differences in EF tests could be caused by the low to moderate power of tests of central tendency (posthoc power analysis: 1 − β = 0.05 (Time A; dTMT) to 0.52 (CBT)).

## 5. Conclusions

The routes in bouldering (climbing up and over rocks at relatively low heights without using ropes but gym mats) or sport climbing can often be climbed in several ways. The athlete has to choose and execute the best possible solution for their skills to climb the route. Additionally, the athletes have to remember this type of “choreography”, which describes a domain-specific cognitive skill. This decision-making and requirement of the climber’s creativity are made possible by higher and more domain-specific cognitive skills [42] but could not be transferred to laboratory tasks. To end up with the question of Voss et al. [2] (p. 813): “Are expert athletes experts in the cognitive laboratory?” The answer regarding the current findings and considering the study’s limitations has to be: “No, the superior cognitive skills of climbers appear to be strongly domain-specific”. The present study results do not implicate that climbing is a sport to support general cognitive or executive functions. Further studies should examine larger sample sizes with significant differences in climbing performance. There could be differences in EF measures between climbers and non-climbers.

## Figures and Tables

**Table 1:** Characteristics of the two groups (novices; experts) of the study population and group differences measured by *t*-test.

	Novices		Experts		*t*	*p*-Value	Cohens *d*
	Mean	SD	M	SD			
Age	25.3	1.6	22.22	4.1	2.072	0.054	1.01
Height	174.1	8.0	174.33	5.8	0.324	0.946	0.01
Weight	69.5	13.2	67.67	9.6	−0.068	0.750	0.08
Training hours/week	1.6	0.5	2.67	1.2	−2.364	0.030 *	−1.19

* = *p* < 0.05.

**Table 2:** Test values for the tests for executive functioning of the two groups (novices; experts). Group differences measured with Mann–Whitney U test.

Test	Parameter	Novices		Experts	
		M	SD	M	SD
Stroop Test	Congruent	731.00	44.94	706.44	59.40
	Incongruent	700.30	44.57	695.11	75.36
	Stroop Effect	−58.30	47.94	−16.67	62.97
CBT	Span	6.50	0.81	5.33	0.82
dTMT	Time (Part A)	35.66	8.64	34.94	4.85
	Time (Part B)	41.35	8.50	41.98	7.59
WCST	Total errors	10.60	2.69	9.33	4.94
	Preservation Errors	6.80	1.66	6.56	2.75
	Non-Preservation Errors	3.80	2.23	2.78	2.48

## Data Availability

The data presented in this study are available on request from the corresponding author.
